# Supplementary material for: Glutaredoxin 1 controls monocyte reprogramming during nutrient stress and protects mice against obesity and atherosclerosis in a sex-specific manner
Source: Nat Commun. 2022 Feb 10;13:790. doi: 10.1038/s41467-022-28433-2 (PMC8831602; doi:10.1038/s41467-022-28433-2)
Supplement: Supplementary file 1 — Supplementary Information [file 41467_2022_28433_MOESM1_ESM.pdf]

Supplementary Information

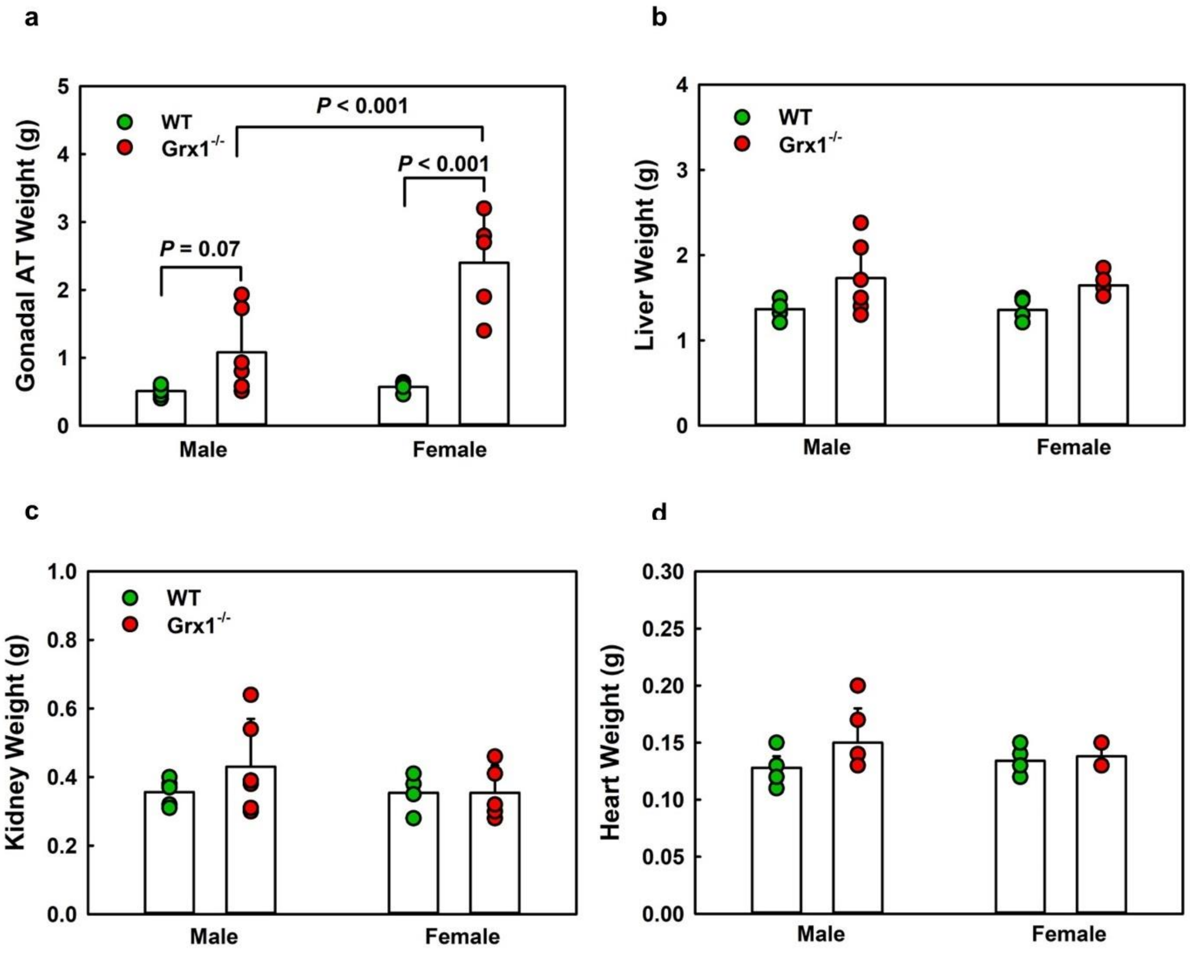

Fig. S1: Grx1 Deficiency Increases Adipose Tissue and Liver Weights in 18-Month-Old Female but not in Male Mice.

(**a**) Gonadal adipose tissue weights

(**b**) Liver weights

(**c**) Kidney weights

(**d**) Heart weights

All data are expressed as mean ± S.D., n = 5 – 6 mice per group for **a-d.** One-way ANOVA followed by Fisher's Least Significance Difference test were used to compare the mean values between experimental groups. Source data are provided as a Source Data file.

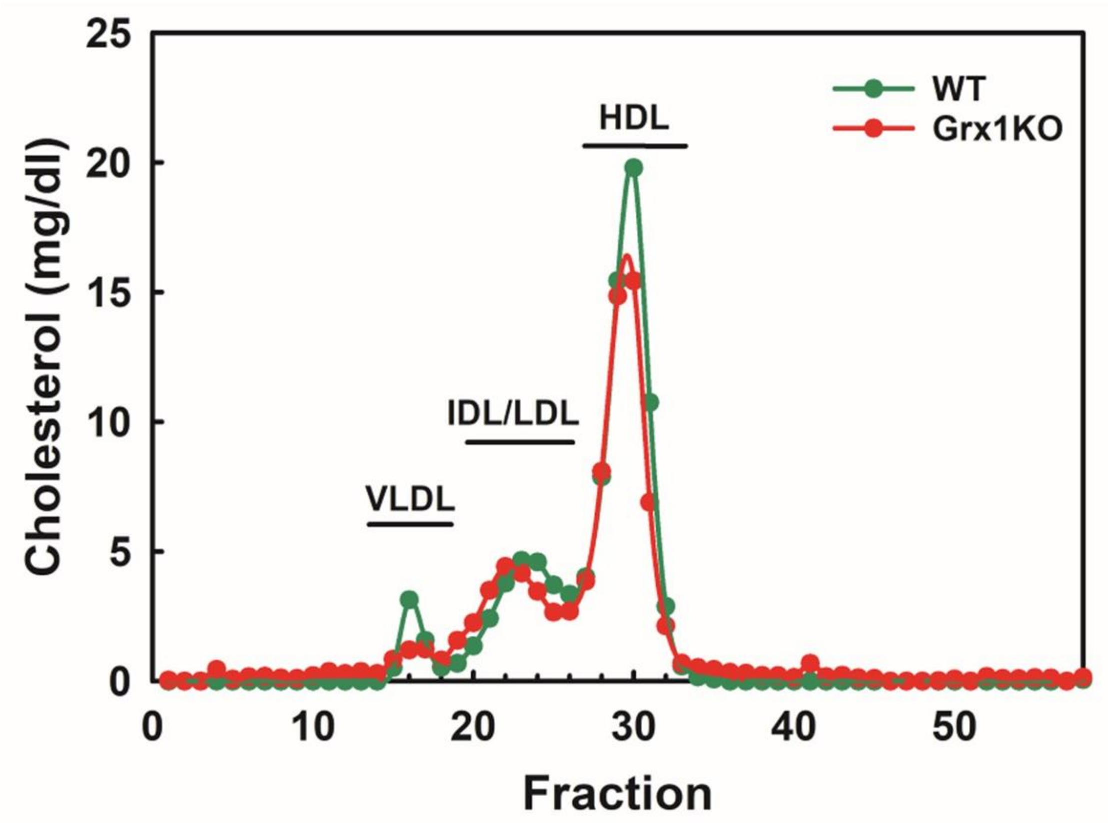

Fig. S2: Grx1 Deficiency Does Not Alter Lipoprotein Profiles in 18-Month-Old Male Mice.

Lipoprotein profiles were generated from pooled plasma samples from aged male Grx1$^{-/-}$ mice (n=3) and age-matched male C57BL/6J mice (n=5). Source data are provided as a Source Data file.

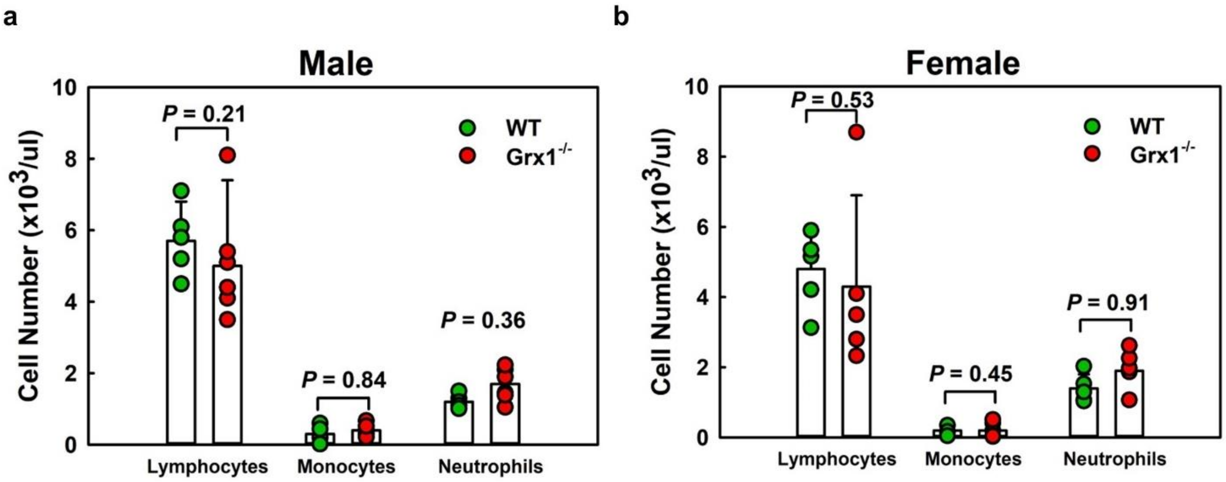

Fig. S3: Grx1 Deficiency Does not Alter Leukocyte Counts of 18-Month-Old Mice.

(**a, b**) Leukocyte counts in male and female Grx1$^{-/-}$ mice and age-matched male and female C57BL/6J mice (WT) maintained on a normal chow diet for 18 months. All data are expressed as mean ± S.D., n=5-6 mice per group. One-way ANOVA followed by Fisher's Least Significance Difference test were used to compare the mean values between experimental groups. Source data are provided as a Source Data file.

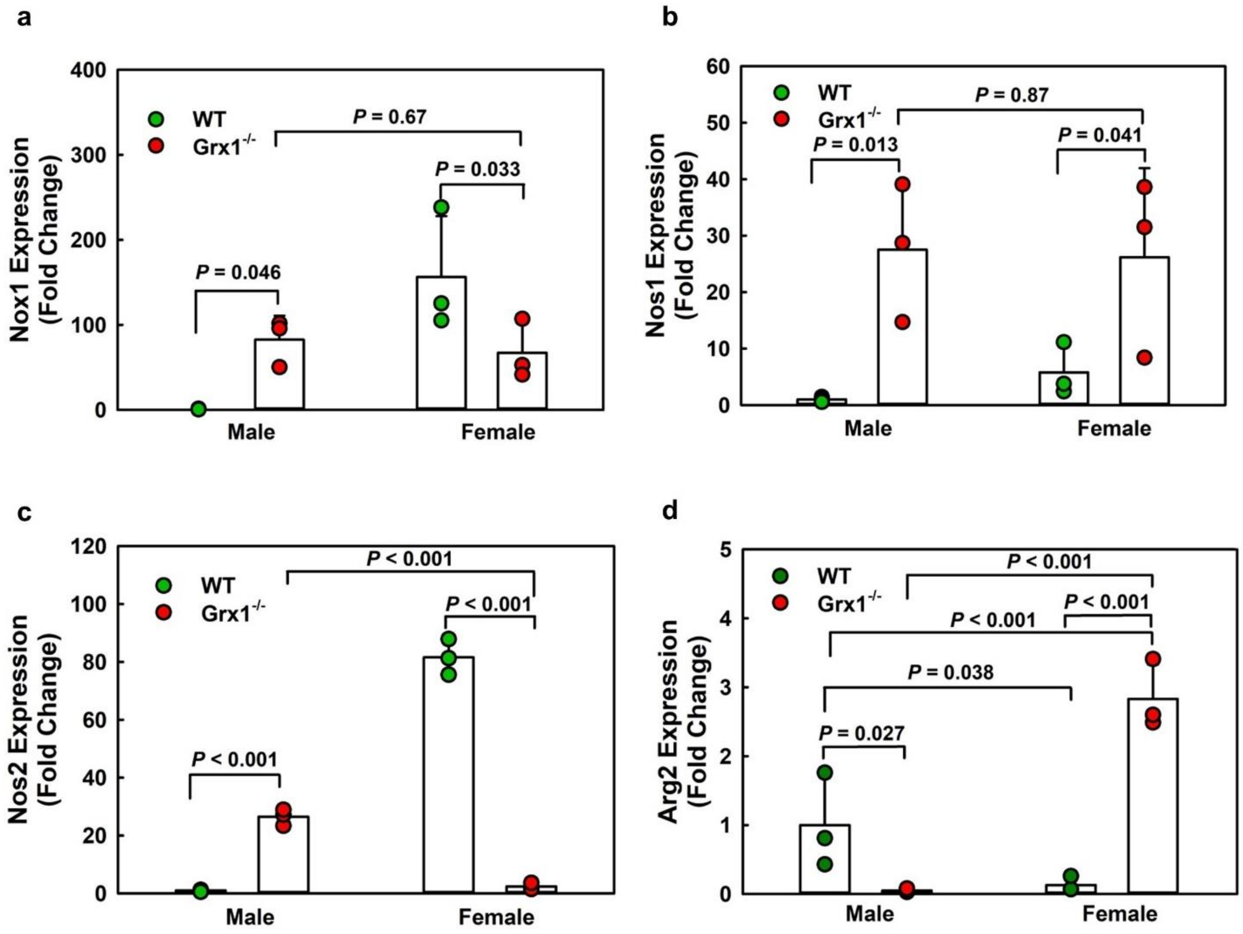

Fig. S4 Macrophage Reprogramming in Aged Grx1-Deficient Mice

(**a**) Derepession of NOX1 expression by Grx1 deficiency in peritoneal macrophages from 18-month-old male but suppression of NOX1 expression in female mice.

(**b**) Derepession of NOS1 expression by Grx1 deficiency in peritoneal macrophages from 18-month-old male and female mice.

(**c**) Derepession of NOS2 expression by Grx1 deficiency in peritoneal macrophages from 18-month-old male but not female mice.

(**d**) Suppression of ARG2 expression by Grx1 deficiency in peritoneal macrophages from 18-month-old male mice and derepression of ARG2 in female mice.

All data are expressed as mean ± S.D, n=3 mice per group. One-way ANOVA followed by Fisher's Least Significance Difference test was used to compare the mean values between experimental groups. Source data are provided as a Source Data file.

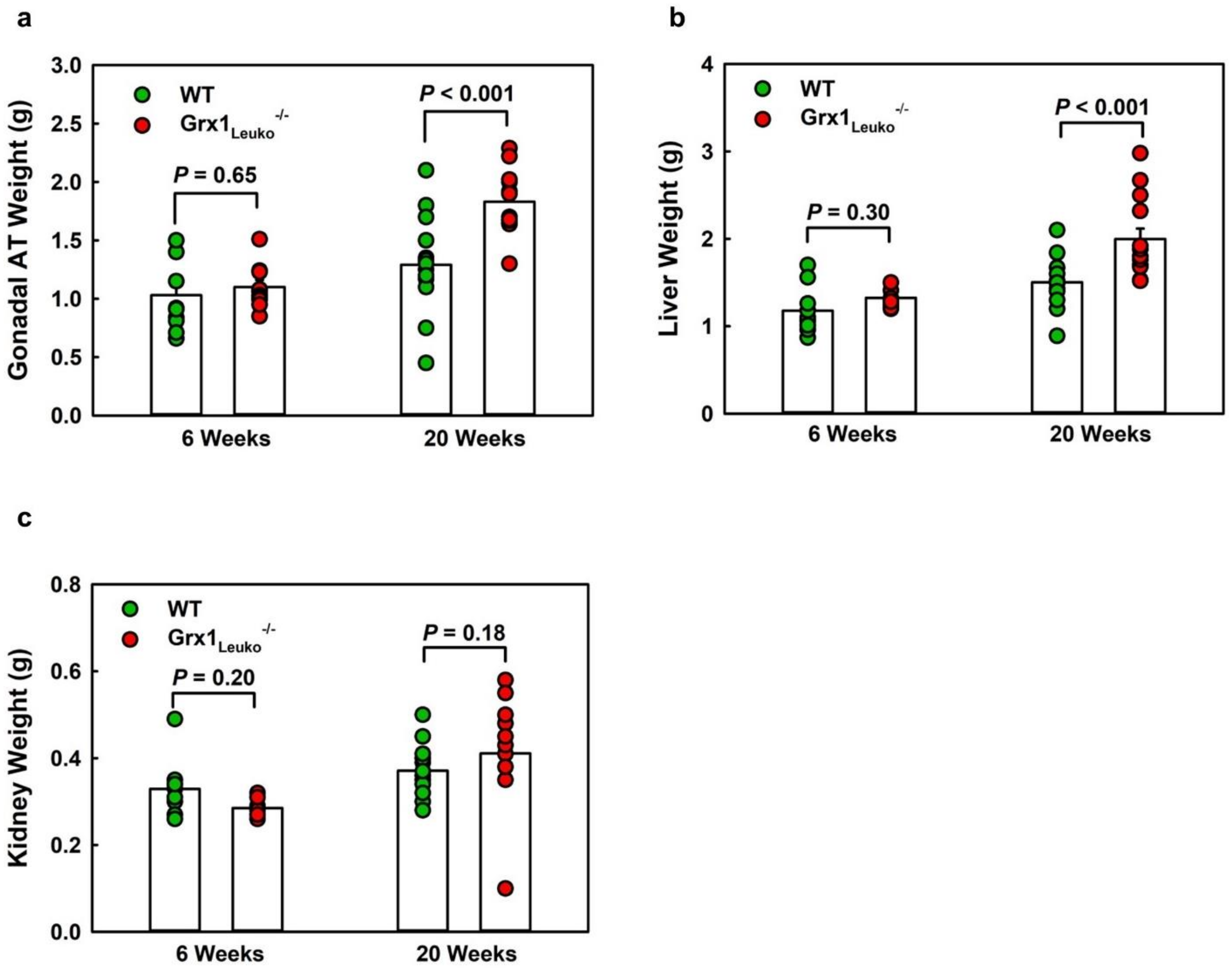

Fig. S5 Hematopoietic Grx1 Deficiency Increases Adipose Tissue and Liver Weights in Reproductively Healthy Female LDLR$^{-/-}$ Mice Fed a HCD for 20 Weeks.

(**a**) Gonadal adipose tissue weights

(**b**) Liver weights

(**c**) Kidney weights

All data are expressed as mean ± S.E., n=14 mice per group. One-way ANOVA followed by Fisher’s Least Significance Difference test were used to compare the mean values between experimental groups. Source data are provided as a Source Data file.

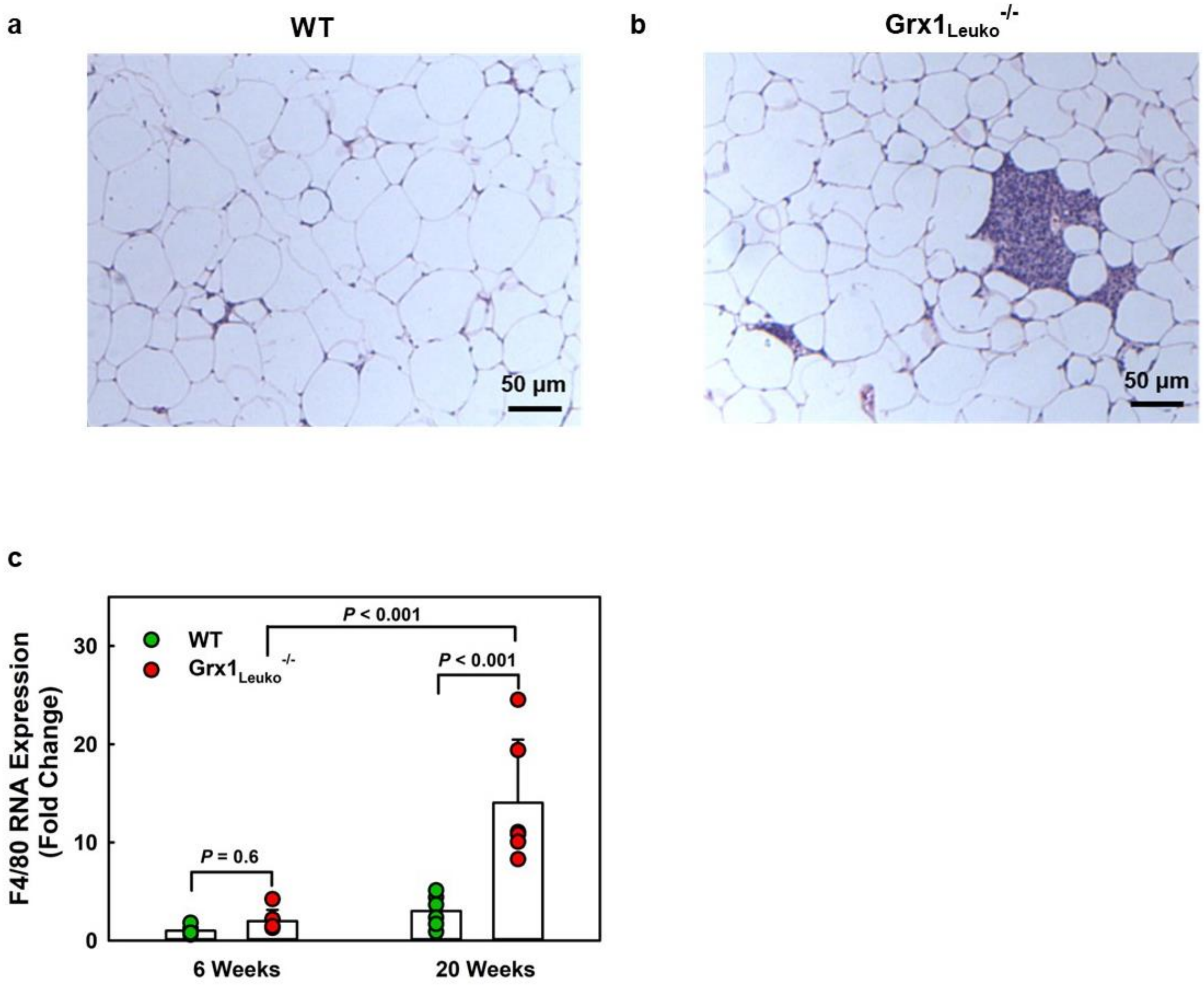

Fig. S6 Hematopoietic Grx1 Deficiency Accelerates Macrophage Infiltration into Gonadal Adipose Tissue in Young Female LDLR$^{-/-}$ mice Fed a HCD for 20 Weeks.

(**a, b**) Representative images of gonadal adipose tissue section stained with H&E.

(**c**) F4/80 mRNA expression in gonadal adipose tissues. All data are expressed as mean ± S.D., n=6 mice per group. One-way ANOVA followed by Fisher's Least Significance Difference test were used to compare the mean values between experimental groups. Source data are provided as a Source Data file.

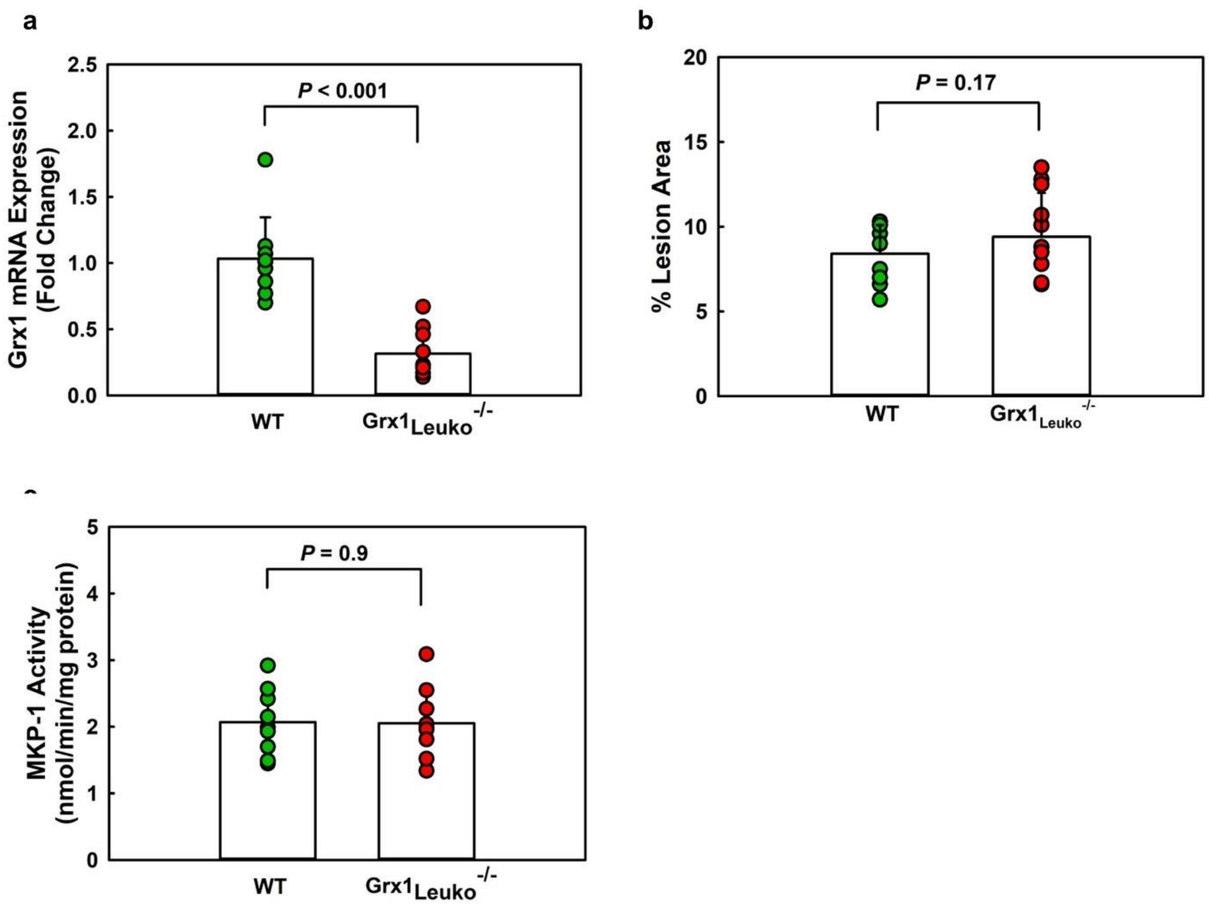

Fig. S7 Hematopoietic Grx1 Deficiency Has no Effect on Atherogenesis in Young Male LDLR$^{-/-}$ mice fed a HCD for 20 Weeks.

(**a**) Grx1 mRNA expression levels in blood monocytes isolated from HCD-fed male LDLR$^{-/-}$Grx1$_{Leuko}^{-/-}$ mice (Grx1$_{Leuko}^{-/-}$) and age-matched female LDLR$^{-/-}$ mice (WT).

(**b**) Quantitation of aortic lesion area in aortas isolated from HCD-fed male LDLR$^{-/-}$Grx1$_{Leuko}^{-/-}$ mice (Grx1$_{Leuko}^{-/-}$) and age-matched male LDLR$^{-/-}$ mice (WT).

(**c**) MKP-1 activity in purified peritoneal macrophages isolated from HCD-fed male LDLR$^{-/-}$Grx1$_{Leuko}^{-/-}$ mice (Grx1$_{Leuko}^{-/-}$) and age-matched male LDLR$^{-/-}$ mice (WT).

All data are expressed as means ± S.D., n=9-10 mice per group for **a-c**. n=9-10 per group. The mean values between experimental groups were compared using the t-test. Source data are provided as a Source Data file.

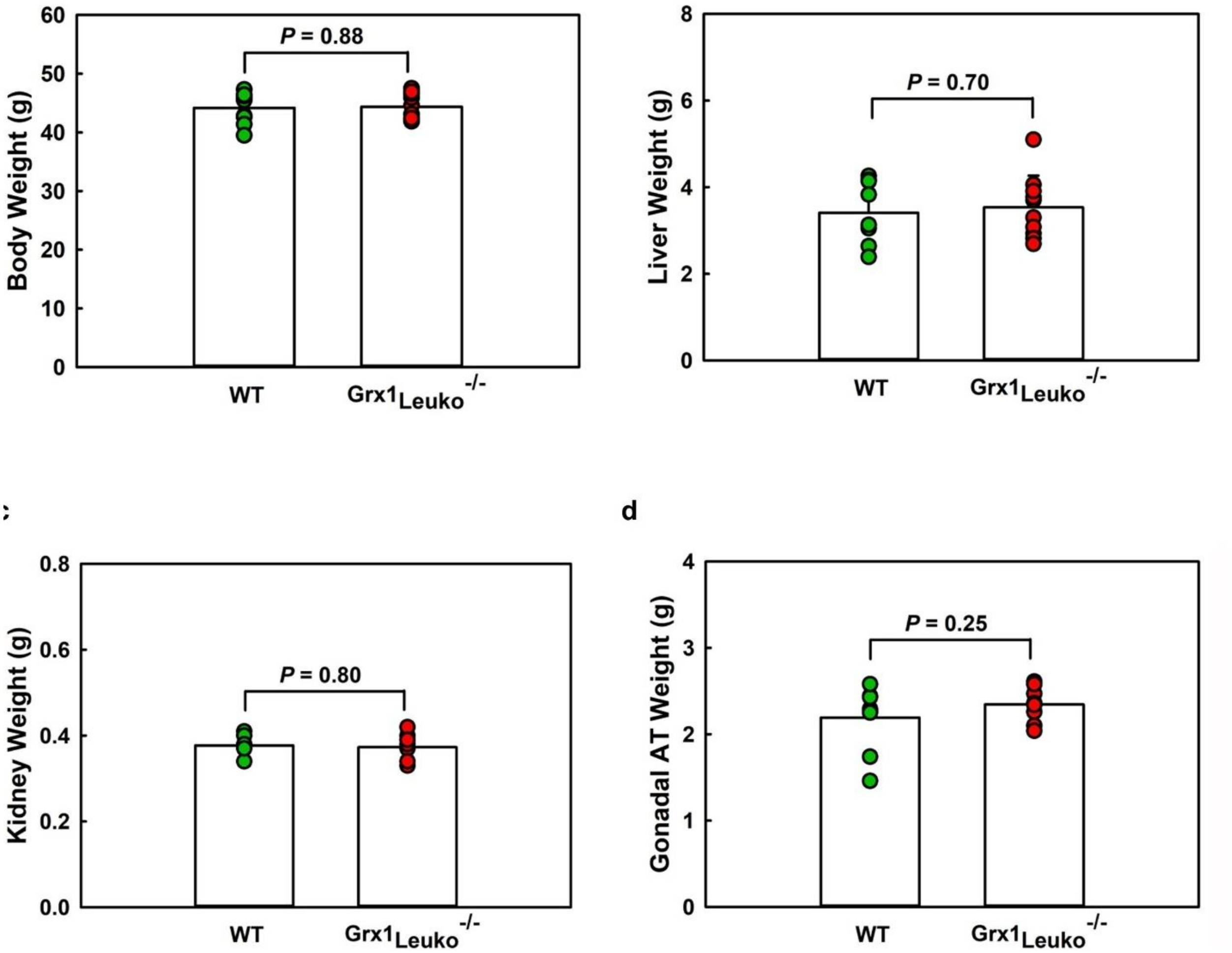

Fig. S8 Hematopoietic Grx1 Deficiency has no Effect on Body or Organ Weights in Young Male LDLR$^{-/-}$ Mice Fed a HCD for 20 Weeks.

(**a**) Body weights

(**b**) Liver weights

(**c**) Kidney weights

(**d**) Gonadal adipose tissue weights

All data are expressed as means ± S.D., n=10 mice per group for **a-d**. The mean values between experimental groups were compared using the t-test. Source data are provided as a Source Data file.

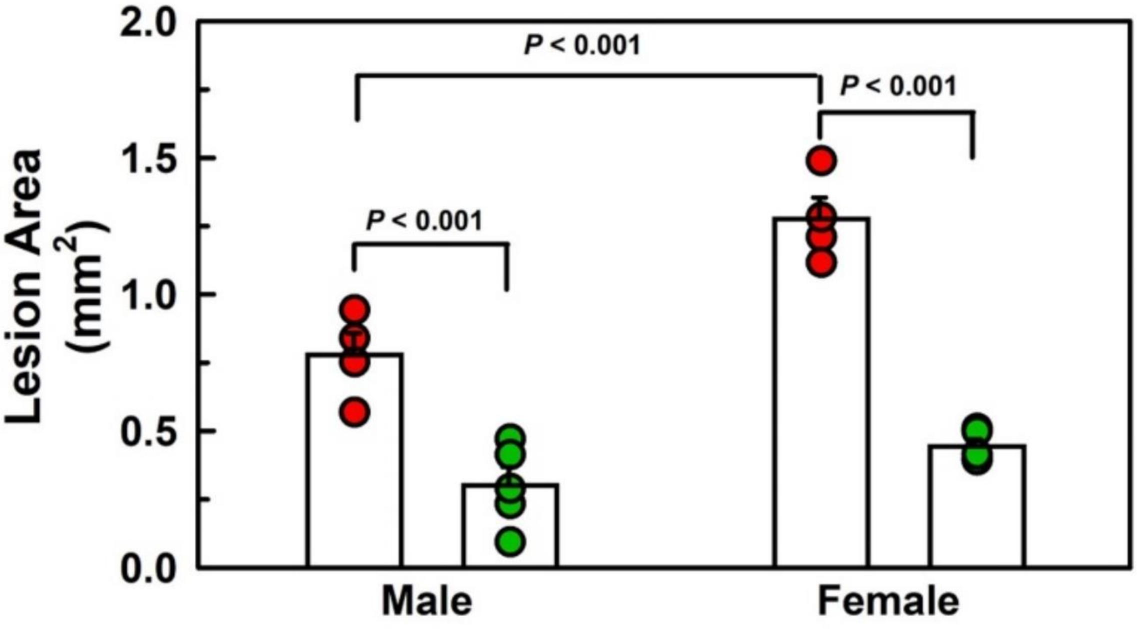

Fig. S9 Macrophage-Restricted Overexpression of Grx1 in LDLR$^{-/-}$ Mice Suppresses HCD Feeding-Induced Atherosclerotic Plaque Formation in Bothe Males and Females.

Quantitation of lesion area in the aortic root of 30-week-old male and female HCD-fed LDLR$^{-/-}$ mice overexpressing either EGFP (red circles) or Grx1 (green circles). All data are expressed as mean ± S.D. n=4 – 6 mice per group. One-way ANOVA followed by Fisher Least Significance Difference test were used to compare the mean values between experimental groups. Source data are provided as a Source Data file

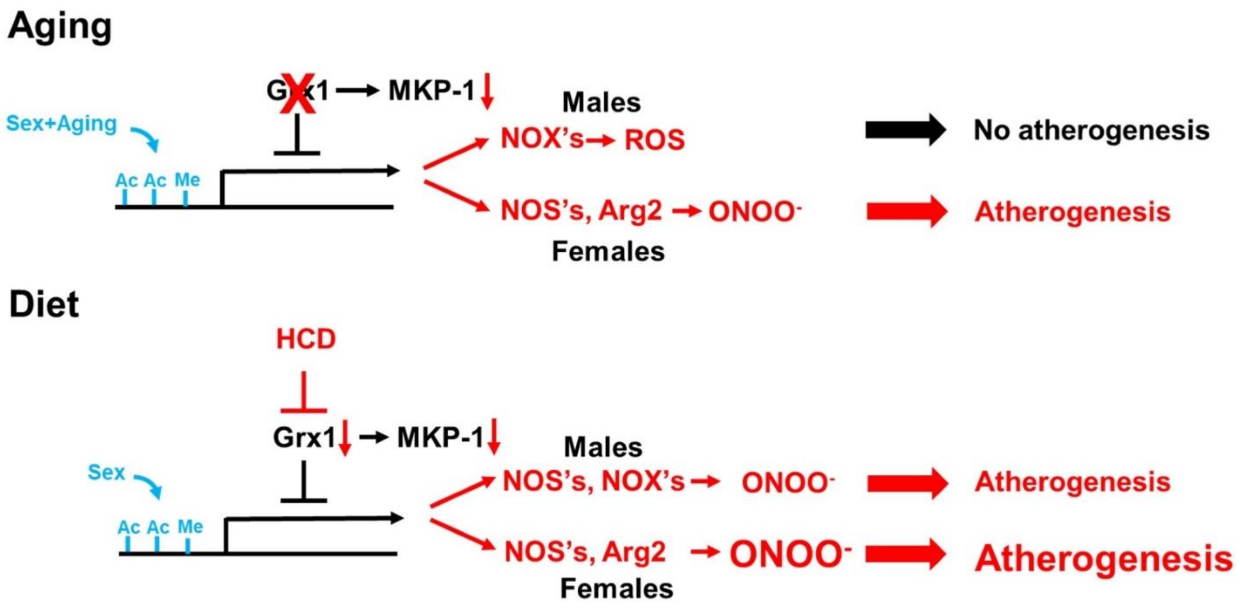

Fig. S10 Loss of Glutaredoxin 1 Activity Leads to Sexual Dimorphic Derepression of NADPH Oxidases and Nitric Oxide Synthases, Reprogramming of Macrophages, Peroxynitrite Formation and Atherogenesis.

The epigenetic landscape (Ac; Me) generated in mice by sex and aging predetermines the expression patterns of ROS and RNS-generating enzymes induced in macrophages either by genetic deletion of glutaredoxin 1 (Grx1) or by high calorie diet (HCD)-induced loss of glutaredoxin 1 activity. These sex-specific expression patterns in turn determine the type of ROS and extent of peroxynitrate ($ONOO^-$) generated by macrophages in these mice and the rate of atherogenesis.

Parameter		Male			Female		
		**WT**	**Grx1$^{-/-}$**	***P***	**WT**	**Grx1$^{-/-}$**	***P***
WBCs	(x 10^3/µl)	7.2 ± 1.4	8.7 ± 2.3	0.27	7.2 ± 0.5	5.2 ± 1.1	0.18
RBCs	(x 10^6/µl)	8.0 ± 0.3	8.9 ± 11.5	0.26	8.0 ± 2.2	8.0 ± 1.0	0.96
Platelets	(x 10^3/µl)	1090 ± 106	1286 ± 344	0.31	962 ± 49	1031 ± 353	0.42

Supplemental Table 1. CBC Analysis for 18-month-old WT and Grx1$^{-/-}$ mice

CBC were analyzed in WT (n=5) and Grx1$^{-/-}$ mice (n=6) at 18 months of age. Mean ± SD. Source data are provided as a Source Data file.

Metabolic Pathways	Gene Symbol	Assay ID
Glycolysis	*Aldoa*	Mm00833172_g1
	Aldoc	Mm01298116_g1
	Bpgm	Mm00500291_m1
	Eno1	Mm01619597_g1
	Eno2	Mm00469062_m1
	Eno3	Mm00468267_m1
	Galm	Mm01233311_m1
	Gapdh	Mm99999915_g1
	Gpi1	Mm01962484_u1
	Hk1	Mm00439344_m1
	Hk2	Mm00443385_m1
	Hk3	Mm01341942_m1
	Ldha	Mm01612132_g1
	Pfkl	Mm00435587_m1
	Pfkm	Mm01309576_m1
	Pfkp	Mm00444792_m1
	Pfkfb2	Mm00435575_m1
	Pfkfb3	Mm00504650_m1
	Pfkfb4	Mm00557176_m1
	Pgam1	Mm02526975_g1
	Pgam2	Mm01187768_m1
	Pgam5	Mm01205823_m1
	Pgk1	Mm00435617_m1
	Pgk2	Mm00446208_s1
	Pkm	Mm00834102_gH
	Tpi1	Mm00833691_g1
Glucose metabolism	*Slc2a1*	Mm00441480_m1
	Slc2a3	Mm00441483_m1
	Slc2a5	Mm00600311_m1
	Slc2a6	Mm00554217_m1
	Slc2a8	Mm00444634_m1
	Slc2a9	Mm00455122_m1
	Slc2a10	Mm01249519_m1
	Slc2a12	Mm02375931_s1
	Slc17a5	Mm00555344_m1
	G6pc	Mm04207416_m1
	Lep	Mm00434759_m1
	Akt1	Mm01331626_m1
	Gcg	Mm01269055_m1
	Pik3r1	Mm01282781_m1

	Lepr	Mm00440181_m1
	Ugp2	Mm00454826_m1
	Pdp1	Mm01217532_m1
	Pdp2	Mm01252669_s1
	Pdhx	Mm00558275_m1
	Pdpr	Mm01243524_m1
	Pdk1	Mm00554300_m1
	Pdk2	Mm00446681_m1
	Pdk3	Mm00455220_m1
	Pdk4	Mm01166879_m1
TCA cycle	*Pck2*	Mm00551411_m1
	Acly	Mm01302282_m1
	Aco1	Mm00801417_m1
	Aco2	Mm00475673_g1
	Cs	Mm00466043_m1
	Dlat	Mm00455160_m1
	Dld	Mm00432831_m1
	Dlst	Mm00513470_m1
	Fh1	Mm01321349_m1
	Idh1	Mm00516030_m1
	Idh2	Mm00612429_m1
	Idh3a	Mm00499674_m1
	Idh3b	Mm00504589_m1
	Idh3g	Mm00599686_g1
	Mdh1	Mm00485106_m1
	Mdh2	Mm00725890_s1
	Ogdh	Mm00803119_m1
	Pdha1	Mm00468675_m1
	Pdhb	Mm00499323_m1
	Sdhc	Mm00481172_m1
	Sdhd	Mm00546511_m1
	Suclg1	Mm00451244_m1
	Suclg2	Mm01182162_g1
Glutamince Metabolism	*Slc1a4*	Mm00444532_m1
	Slc1a5	Mm00436603_m1
	Slc3a2	Mm00500521_m1
	Slc7a5	Mm00441516_m1
	Slc7a6	Mm00626779_m1
	Slc7a7	Mm00448764_m1
	Slc38a1	Mm00506391_m1
	Slc38a2	Mm00628416_m1

	Aldh4a1	Mm00615268_m1
	Aldh18a1	Mm00444767_m1
	Asl	Mm01197741_m1
	Ass1	Mm00711256_m1
	Cad	Mm01216345_m1
	Gfpt1	Mm00600127_m1
	Gfpt2	Mm00496565_m1
	Pfas	Mm01325237_m1
	Ppat	Mm00549096_m1
	Glud1	Mm00492353_m1
	Glul	Mm00725701_s1
	Gls	Mm01257297_m1
	Got1	Mm01195792_g1
	Got2	Mm00494703_m1
	Gpt	Mm00805379_g1
	Gpt2	Mm00558028_m1
	Nit2	Mm00517778_m1
	Oat	Mm00497544_m1
	Odc1	Mm01964631_g1
	Prodh	Mm00448401_m1
	Srm	Mm00726089_s1
	Slc6a12	Mm00446675_m1
Transcription factors	*Nfkb1*	Mm00476361_m1
	Hif1a	Mm00468869_m1
	Epas1	Mm01236112_m1
	Pparg	Mm01184322_m1
	Ppara	Mm00440939_m1
	Ppard	Mm00803184_m1
	Stat1	Mm01257286_m1
	Stat2	Mm00490880_m1
	Stat3	Mm01219775_m1
	Stat4	Mm00448881_m1
	Stat6	Mm01160477_m1
	Klf4	Mm00516104_m1
	Klf6	Mm00516184_m1
	Rxra	Mm00441185_m1
	Rxrg	Mm00436411_m1
	Nr1h2	Mm00437265_g1
Inflammatory markers	*Myd88*	Mm00440338_m1
	Arg2	Mm00477592_m1
	Ccr5	Mm04207879_m1

	Ccr7	Mm01301785_m1
	Ccl8	Mm01297183_m1
	Cd86	Mm00444543_m1
	Cxcl9	Mm00434946_m1
	Cxcl10	Mm00445235_m1
	Cxcl13	Mm04214185_s1
	Il1b	Mm00434228_m1
	Il6	Mm00446190_m1
	Il12b	Mm00434174_m1
	Il31ra	Mm01304494_m1
	Il17ra	Mm00434214_m1
	Ptgs2	Mm00478374_m1
	Rela	Mm00501346_m1
	Tlr2	Mm00442346_m1
	Tlr4	Mm00445273_m1
	Tnf	Mm00443258_m1
	Ifng	Mm01168134_m1
Inflammation resolving	*Arg1*	Mm00475988_m1
	Ccl2	Mm00441242_m1
	Ccl7	Mm00443113_m1
	Ccl9	Mm00441260_m1
	Ccl17	Mm00516136_m1
	Cd36	Mm01135198_m1
	Cd163	Mm00474091_m1
	Mrc1	Mm01329362_m1
	Cd209a	Mm00460067_m1
	Chil3	Mm00657889_mH
	Il10	Mm00439614_m1
	Igf1	Mm00439560_m1
	Folr2	Mm00433357_m1
	Mgl2	Mm00460844_m1
	Mrc1	Mm00485148_m1
	Retnla	Mm00445109_m1
	Tfrc	Mm00441941_m1
	Tgfb1	Mm01178820_m1
	COX1	Mm04225243_g1
	Ppargc1b	Mm00504720_m1
	Tfam	Mm00447485_m1
Deacetylases	*Sirt1*	Mm00490758_m1
	Sirt2	Mm01149204_m1
	Sirt3	Mm00452131_m1

	Sirt4	Mm01201915_m1
	Sirt5	Mm00663723_m1
	Sirt6	Mm01149042_m1
	Sirt7	Mm01248607_m1
	Hdac1	Mm02745760_g1
	Hdac2	Mm00515108_m1
	Hdac3	Mm00515916_m1
	Hdac6	Mm00515945_m1
Antioxidant system	*Gpx1*	Mm00656767_g1
	Gpx2	Mm00850074_g1
	Gpx3	Mm00492427_m1
	Gpx4	Mm00515041_m1
	Gpx7	Mm00481133_m1
	Gpx8	Mm01297261_m1
	Gsr	Mm00439154_m1
	Gsta3	Mm00494798_m1
	Gsta4	Mm00494803_m1
	Gstk1	Mm00504022_m1
	Gstm1	Mm00833915_g1
	Gstm2	Mm00725711_s1
	Gstm4	Mm00728197_s1
	Gstm5	Mm00515890_m1
	Gsto1	Mm00599866_m1
	Gsto2	Mm00509763_m1
	Gstp1	Mm04213618_gH
	Gstt1	Mm00492506_m1
	Gstt2	Mm00494804_g1
	Gstz1	Mm00515900_m1
	Mgst1	Mm00498294_m1
	Mgst2	Mm00723390_m1
	Mgst3	Mm00787806_s1
	Prdx1	Mm01621996_s1
	Prdx2	Mm04208213_g1
	Prdx3	Mm00545848_m1
	Prdx4	Mm00450261_m1
	Prdx5	Mm00465365_m1
	Prdx6	Mm00725435_s1
	Gss	Mm00515065_m1
	Gclc	Mm00802655_m1
	Gclm	Mm00514996_m1
	Glrx2	Mm00469836_m1

	Glrx	Mm00728386_s1
	Sod1	Mm01344233_g1
	Sod2	Mm01313000_m1
	Sod3	Mm01213380_s1
	Cat	Mm00437992_m1
ROS generators	*Nox4*	Mm00479246_m1
	Cybb	Mm01287743_m1
	Nox1	Mm00549170_m1
	Maoa	Mm00558004_m1
	Maob	Mm00555412_m1
	Nos1	Mm01208059_m1
	Nos2	Mm00440502_m1
	Nos3	Mm00435217_m1
Fatty acid oxidation	*Cd36*	Mm00432403_m1
	Acadl	Mm00599660_m1
	Acadvl	Mm00444293_m1
	Acadm	Mm01323360_g1
	Acads	Mm00431617_m1
	Cpt1a	Mm01231183_m1
	Cpt1b	Mm00487200_m1
	Slc27a1	Mm00449511_m1
	Slc27a3	Mm01220017_m1
	Slc27a4	Mm01327405_m1
	Slc27a5	Mm00447768_m1
	Slc27a6	Mm01258609_m1
	Fabp1	Mm00444340_m1
	Fabp2	Mm00433188_m1
	Fabp3	Mm02342495_m1
	Fabp4	Mm00445878_m1
	Me1	Mm00782380_s1
	Me3	Mm00724881_m1
	Sdhb	Mm00458272_m1
	Cpt2	Mm00487205_m1
	Acox1	Mm01246834_m1
	Slc25a20	Mm00451571_m1
	Hsd17b4	Mm00500443_m1
	Alb	Mm00802090_m1
	Scp2	Mm01257982_m1
	Ehhadh	Mm00619685_m1
	Hadha	Mm00805228_m1
	Hadhb	Mm00695255_g1

	Hadh	Mm00492535_m1
Fatty acid synthesis	*Fads1*	Mm00507605_m1
	Fads2	Mm00517221_m1
	Fads3	Mm01200850_m1
	Fads6	Mm00626150_m1
	Scd1	Mm00772290_m1
	Scd2	Mm01208542_m1
	Scd4	Mm01208549_m1
	Degs1	Mm00492146_m1
	Foxred1	Mm00549438_m1
	Foxred2	Mm01720535_m1
	Rnls	Mm04178677_m1
	Ndor1	Mm00626390_m1
	Acsf3	Mm00460721_m1
	Ndufab1	Mm01137654_g1
	Dgat1	Mm00515643_m1
	Dgat2	Mm00499536_m1
	Fasn	Mm00662319_m1
Cholesterol metabolism	*Hmgcs1*	Mm01304569_m1
	Hmgcr	Mm01282499_m1
	Pmvk	Mm01212763_m1
	Fdft1	Mm01598574_g1
	Mvk	Mm00445773_m1
	Fdps	Mm00836315_g1
	Dhcr7	Mm00514571_m1
	Lss	Mm00461312_m1
	Sqle	Mm00436772_m1
	Ggps1	Mm00656129_mH
	Ndufa1	Mm00444593_m1
	Ndufa2	Mm00477755_g1
	Ndufa3	Mm01329704_g1
	Ndufa4	Mm00809672_s1
	Ndufa5	Mm01165335_m1
	Ndufa6	Mm01303455_g1
	Ndufa7	Mm00458227_m1
	Ndufa8	Mm00503351_m1
	Ndufa9	Mm00481216_m1
	Ndufa10	Mm00600325_m1
	Ndufa11	Mm01236867_g1
	Ndufa12	Mm01240336_m1
	Acat1	Mm00507463_m1

	Acat2	Mm00782408_s1
	Acat3	Mm00462484_m1
	Acaca	Mm01304257_m1
	Acacb	Mm01204671_m1
	Srebf1	Mm00550338_m1
	Plin2	Mm00475794_m1
	Etfdh	Mm01220210_m1
	Apoa1	Mm00437569_m1
	Apoa2	Mm00442687_m1
	Apob	Mm01545159_m1
	Apoc1	Mm00431816_m1
	Apoc2	Mm00437571_m1
	Lcat	Mm01247340_m1
	Lpl	Mm00434764_m1
	Npc1	Mm00435300_m1
	Mttp	Mm00435015_m1
	Npc2	Mm00499230_m1
	Angptl3	Mm00803820_m1
ATP-binding cassette transporters	*Abcc1*	Mm00456156_m1
	Abcc3	Mm00551550_m1
	Abcc4	Mm01226381_m1
	Abcc5	Mm01343626_m1
	Abcc8	Mm00803450_m1
	Abcc10	Mm00467403_m1
	Abca1	Mm00442646_m1
MKPs	*Dusp1*	Mm00457274_g1
	Prickle2	Mm01182431_m1
	Dusp6	Mm00518185_m1
	Dusp9	Mm00512648_g1
MAPKs	*Mapk8*	Mm00489514_m1
	Mapk14	Mm01301009_m1
AMPKs	*Prkaa1*	Mm01296700_m1
	Prkaa2	Mm01264789_m1
	Prkab1	Mm01201921_m1
	Prkab2	Mm01257133_m1
	Prkag1	Mm00450298_g1
	Prkag2	Mm00513977_m1
PKCs	*Prkca*	Mm00440858_m1
	Prkcb	Mm00435749_m1
	Prkcg	Mm00440861_m1
	Prkcd	Mm00440891_m1

	Prkce	Mm00440894_m1
	Prkci	Mm00435769_m1
	Prkcq	Mm01340228_m1

Supplemental Table 2. Lists of Genes Selected for TaqMan® Assay Card

Parameter		6 weeks			20 weeks		
		**WT**	**Grx1$_{Leuko}^{-/-}$**	***P***	**WT**	**Grx1$_{Leuko}^{-/-}$**	***P***
WBCs	(x 10^3/µl)	4.7 ± 0.7	3.8 ± 0.6	0.367	5.1 ± 0.6	4.0 ± 0.4	0.168
RBCs	(x 10^6/µl)	8.9 ± 0.2	8.8 ± 0.2	0.786	8.3 ± 0.3	8.3 ± 0.2	0.991
Platelets	(x 10^3/µl)	372 ± 30	402 ± 26	0.467	277 ± 51	415 ± 69	0.117
Lymphocytes	%	80.5 ± 2.1	88.7 ± 1.2	**0.003**	74.1 ± 4.1	65.1 ± 6.4	0.127
Monocytes	%	3.4 ± 0.9	3.3 ± 0.6	0.946	4.9 ± 0.9	3.7 ± 0.7	0.326

Supplemental Table 3. CBC Analysis for female WT and Grx1$_{Leuko}^{-/-}$ Mice

CBC analysis from female WT or Grx1$_{Leuko}^{-/-}$ mice fed a HCD for 6 and 20 weeks. Mean ± SEM. Source data are provided as a Source Data file.

	Symbol	Gene Name	Assay ID
Proinflammatory Polarization ($M\Phi_{IFN\text{-}\gamma+TNF\text{-}\alpha}$)	*Tnfα*	Tumor necrosis factor	Mm00443258_m1
	Il1β	Interleukin 1 beta	Mm00434228_m1
	Il6	Interleukin 6	Mm00446190_m1
	Il17ra	Interleukin 17 receptor A	Mm00434214_m1
	Ccl2	Chemokine (C-C motif) ligand 2	Mm00441242_m1
	Cxcl9	Chemokine (C-X-C motif) ligand 9	Mm00434946_m1
	Cd86	CD86 antigen	Mm00444543_m1
Inflammation Resolving ($M\Phi_{IL\text{-}4}$)	*Il10*	Interleukin 10	Mm01288386_m1
	Tgfβ1	Transforming growth factor beta 1	Mm01178820_m1
	Ccl7	Chemokine (C-C motif) ligand 7	Mm00443113_m1
	Cd163	CD163 antigen	Mm00474091_m1
	Tfrc	Transferrin receptor	Mm00441941_m1
	Cd36	CD36 antigen	Mm00432403_m1
	Igf1	Insulin-like growth factor	Mm00439560_m1
	Ym1 (Chi3l3)	Chitinase-like 3	Mm00657889_mH

Supplemental Table 4. TaqMan Primers Used for Macrophage Activation Profiling.
