# Supplementary material for: Glutaredoxin 1 controls monocyte reprogramming during nutrient stress and protects mice against obesity and atherosclerosis in a sex-specific manner
Source: Nat Commun. 2022 Feb 10;13:790. doi: 10.1038/s41467-022-28433-2 (PMC8831602; doi:10.1038/s41467-022-28433-2)
Supplement: Supplementary file 3 — Reporting Summary [file 41467_2022_28433_MOESM3_ESM.pdf]

nature research

Corresponding author(s): R. Asmis

Last updated by author(s): Jan 3, 2022

Reporting Summary

Nature Research wishes to improve the reproducibility of the work that we publish. This form provides structure for consistency and transparency in reporting. For further information on Nature Research policies, see Authors & Referees and the Editorial Policy Checklist.

Statistics

For all statistical analyses, confirm that the following items are present in the figure legend, table legend, main text, or Methods section.

n/a	Confirmed	
☐	☒	The exact sample size (*n*) for each experimental group/condition, given as a discrete number and unit of measurement
☐	☒	A statement on whether measurements were taken from distinct samples or whether the same sample was measured repeatedly
☐	☒	The statistical test(s) used AND whether they are one- or two-sided<br>*Only common tests should be described solely by name; describe more complex techniques in the Methods section.*
☒	☐	A description of all covariates tested
☐	☒	A description of any assumptions or corrections, such as tests of normality and adjustment for multiple comparisons
☐	☒	A full description of the statistical parameters including central tendency (e.g. means) or other basic estimates (e.g. regression coefficient) AND variation (e.g. standard deviation) or associated estimates of uncertainty (e.g. confidence intervals)
☐	☒	For null hypothesis testing, the test statistic (e.g. *F*, *t*, *r*) with confidence intervals, effect sizes, degrees of freedom and *P* value noted<br>*Give P values as exact values whenever suitable.*
☒	☐	For Bayesian analysis, information on the choice of priors and Markov chain Monte Carlo settings
☒	☐	For hierarchical and complex designs, identification of the appropriate level for tests and full reporting of outcomes
☒	☐	Estimates of effect sizes (e.g. Cohen's *d*, Pearson's *r*), indicating how they were calculated

Our web collection on statistics for biologists contains articles on many of the points above.

Software and code

Policy information about availability of computer code

Data collection	NICORN 7.6 (FPLC), Carestream V 5.4.2 (Western Blot, Chemotaxis assay), cellSens 2.3 (Images), QuantStudio Real-Time PCR Software v1.3 (qPCR), SoftMax Pro 5.2 (Plate reader), Cellometer Vision 5 (cell counter)
Data analysis	SSigmaPlot 14, JMP Genomics 9, Morpheus (Resource ID: SCR_017386, https://software.broadinstitute.org/morpheus [software.broadinstitute.org]), and Carestream V5.4.2, Image Pro Plus (Image analysis)

For manuscripts utilizing custom algorithms or software that are central to the research but not yet described in published literature, software must be made available to editors/reviewers. We strongly encourage code deposition in a community repository (e.g. GitHub). See the Nature Research guidelines for submitting code & software for further information.

Data

Policy information about availability of data

All manuscripts must include a data availability statement. This statement should provide the following information, where applicable:

- Accession codes, unique identifiers, or web links for publicly available datasets
- A list of figures that have associated raw data
- A description of any restrictions on data availability

Data available on request from the authors.

Field-specific reporting

Please select the one below that is the best fit for your research. If you are not sure, read the appropriate sections before making your selection.

☒ Life sciences ☐ Behavioural & social sciences ☐ Ecological, evolutionary & environmental sciences

Life sciences study design

All studies must disclose on these points even when the disclosure is negative.

Sample size	Sample size for the animal experiments were determined based on power calculation which in turn were based on our previous published atherosclerosis experiments and anticipated changes in atherosclerosis lesion size. We anticipated an increase in atherosclerotic lesions of 25% by power calculation. For the 18 month aging study, we started with five males and five females in each group.
Data exclusions	Two mice from bone marrow transplantation were excluded due to deteriorating health condition (weight loss and death) during high-calorie diet feeding. We used Western blotting to reconfirm the genotype of the aging Grx1-/- mice and and had to exclude two males and one female Grx1-/- mice (which in fact were heterozygous) which previously were originally genotyped twice by PCR and identified as knockout mice. The data from those mice were excluded.
Replication	Protein assays, chemotaxis assays, and MKP-1 assays always included technical duplicates. In cases were not all aniumals in a groupd were used for analysis, the animals were randomly selected.<br>All in vitro cell culture experiment were repeated at least three times, and each experiment included three technical replicates.
Randomization	For bone marrow transplantation, the recipient mice were randomly selected for injection of either WT or Grx1-/- bone marrow.
Blinding	The investigators were blinded with regard to sampling, assay execution and the data collection.

Reporting for specific materials, systems and methods

We require information from authors about some types of materials, experimental systems and methods used in many studies. Here, indicate whether each material, system or method listed is relevant to your study. If you are not sure if a list item applies to your research, read the appropriate section before selecting a response.

Materials & experimental systems

n/a	Involved in the study	
☐	☒	Antibodies
☐	☒	Eukaryotic cell lines
☒	☐	Palaeontology
☐	☒	Animals and other organisms
☒	☐	Human research participants
☒	☐	Clinical data

Methods

n/a	Involved in the study	
☒	☐	ChIP-seq
☒	☐	Flow cytometry
☒	☐	MRI-based neuroimaging

Antibodies

Antibodies used	Caspase 3 (1:1000, Cell Signaling, Cat.#9662, clone: polyclonal antibody from purified serum, Lot# 18), cleaved Caspase-3 (1:1000, Cell Signaling, Cat. # 9661, clone Aspl 75, Lot.# 42), PARP (1:1000, Cell signaling, Cat. #9542, clone: polyclonal antibody from purified serum, Lot# 14), beta-actin antibody (1:1000, Cell Signaling, Cat.# 4970, clone: 13E5, Lot#15). CD68 antibody (1:200, Biorad, Cat.# MCA1957, clone FA-11, Lot# 151603A), Rat IgG2a (1:200, Biorad, Cat.# MCA1212, clone: YTH71.3, Lot#149141). Anti-nitrotyrosine antibody (1:500, EMD Millipore, Cat.# AB5411, polyclonal antibody, Lot# 3474160).
Validation	The data are provided in the manuscript. Relevant references for Caspase-3 and PARP antibodies used in macrophages are PMID 29844306 and PMID 23598404. CD68 antibody was verified by Biorad for immunohistology-frozen and PMID 19592463. IgG2a was verified in PMID 22759398. Anti-nitrotyrosine antibody was verified in PMID 23127860.

Eukaryotic cell lines

Policy information about cell lines

Cell line source(s)	HEK293T cells obtained from ATCC (CRL-3216).
Authentication	HEK293T cell line used was not authenticated.
Mycoplasma contamination	The cell lines were not tested for mycoplasma contamination after they arrived from ATCC.
Commonly misidentified lines (See ICLAC register)	N/A

Animals and other organisms

Policy information about studies involving animals; ARRIVE guidelines recommended for reporting animal research

Laboratory animals	Mouse, C57BL6/J (WT, males and females, 18-month-old), Grxl-/- (males and females, 18-month-old), LDL-R-/-((B6.129S7-Ldlrtm1Her/J, male and female mice, 10 weeks at start, 34 weeks old at endpoint (4 weeks on maintenance diet and recovery after irradiation and 20 weeks on HCD)
Wild animals	This study does not involve wild animals.
Field-collected samples	This study does not involve samples collected from the field.
Ethics oversight	This study were performed in accordance with the guidelines and regulations of and with the approval of the University of Texas Health at San Antonio and Wake Forest School of Medicine Institutional Animal Care and Use Committees.

Note that full information on the approval of the study protocol must also be provided in the manuscript.
